# Decision‐Making Process of Healthcare Providers Regarding Catheterization Method: A Nationwide Survey Study

**DOI:** 10.1002/nau.70215

**Published:** 2026-01-29

**Authors:** Coen Huibert Harry Christiaans, Felice Emanuela Espèrance van Veen, Jeroen Ronald Scheepe, Bertil Freddo Maarten Blok

**Affiliations:** ^1^ Department of Urology Erasmus MC Rotterdam Rotterdam The Netherlands

**Keywords:** clean intermittent catheterization, indwelling catheters, medical decision making, shared decision making, urinary catheterization

## Abstract

**Background:**

The decision on which catheterization method to prescribe should be made on an individual basis, considering each patient's individual needs and circumstances. However, the current decision‐making process regarding assisted bladder drainage might not be transparent or standardized.

**Objectives:**

The aim of the present study was to explore and compare the decision‐making processes of Dutch healthcare providers regarding the choice of catheterization method and relevant bladder management. This information is crucial in the empowerment of patient involvement and the development of a catheter decision aid.

**Design & Methods:**

We conducted a nationwide survey study including urologists, rehabilitation doctors, physician assistants, and specialized (continence)nurses. A 12‐question survey was distributed regarding the decision‐making process, including questions about treatment options discussed and factors upon which healthcare providers base their decisions.

**Results:**

A total of 108 healthcare providers responded (response rate 36%). The majority were (continence)nurses or urologists and worked in a hospital. (Continence)nurses were least often involved in the decision‐making, and when involved, 53% did not discuss potential other treatment options for the underlying causes of impaired bladder emptying. Most healthcare providers base their decision on the patient characteristics.

**Conclusion:**

We observed differences in the decision‐making process between the healthcare providers. Implementing shared decision‐making can lead to more effective collaboration between the patient and healthcare provider when selecting the most appropriate type of bladder management. This could be achieved through comprehensive training supplemented by a validated decision aid.

## 1 Introduction

Urinary catheters are crucial devices in the management of urinary retention or post‐void residual volume [1]. The primary objective is timely bladder drainage to prevent complications such as urinary tract infections, incontinence, or renal insufficiency. In the management of urinary retention, both indwelling catheters (IDCs), transurethral or suprapubic, and clean intermittent catheterization (CIC) are used. According to the professional guidelines, CIC is preferred over an IDC because it is thought to reduce the risk of catheter‐associated urinary tract infections, bladder stones, discomfort, and renal deterioration [2, 3]. Consequently, CIC increases the quality of life (QoL) through greater independence, mobility, and maintaining the ability to engage in sexual activity [3, 4]. However, performing CIC requires sufficient manual dexterity and cognitive function [5].

IDCs are preferably only used in patients unwilling or unable to perform CIC [6]. Furthermore, IDCs are used in the management of urinary incontinence. In patients with (severe) incontinence or with a disability which makes it difficult to use the bathroom, IDCs can increase the QoL [7].

Both urinary retention and incontinence can be neurogenic or non‐neurogenic in origin. Neurogenic causes include spinal cord injury, spina bifida, Parkinson's disease, and multiple sclerosis [8]. Non‐neurogenic causes include bladder outlet obstruction (benign prostate hyperplasia, urethral stricture), post‐pelvic surgery retention, or idiopathic [9]. Lower urinary tract symptoms associated with these causes are often managed by urologists, rehabilitation doctors, physician assistants (PAs), and specialized (continence)nurses in hospitals and rehabilitation centers.

Studies examining patient satisfaction and QoL across different types of catheterization have yielded varied results. Furthermore, the majority of these studies focus on patients with neurogenic bladder dysfunction, which may limit the generalizability of the findings to non‐neurogenic populations [10, 11, 12].

Ultimately, the decision on which type of catheterization or other bladder management to prescribe should be made on an individual basis, considering each patient's individual needs and circumstances. However, the current decision‐making process regarding assisted bladder drainage might not be transparent or standardized [13]. The choice of a specific catheter or discussion of alternative treatment options depends on the preference of the healthcare provider, which is usually based on clinical experience and available treatment options combined with the acquaintance of specific manufacturers. A validated urinary catheter decision aid could help standardize the decision‐making process and increase patient involvement, ultimately improving the standard of care.

The aim of the study was to explore and compare the decision‐making processes of Dutch healthcare providers regarding the choice of catheterization method and relevant bladder management. This information is crucial in empowering patients' involvement and the development of a catheter decision aid.

## 2 Design and Methods

### 2.1 Selection of Healthcare Providers

A nationwide survey study was conducted from August to September 2024. Participants in this study included healthcare providers involved in the care of patients who perform CIC or have an IDC. The participants composed of urologists (in training), (continence)nurses (in training), PAs (in training), and rehabilitation doctors (in training). We requested the 12 hospitals collaborating in the reusable catheter COMPaRE‐trial (trial registration number: NL8296) to distribute the questionnaire to their healthcare providers. Additionally, all 14 rehabilitation clinics in the Netherlands were asked to distribute the questionnaire to their healthcare providers. This study was approved by the Local Medical Ethical Review Committee (MEC‐2024‐0304).

### 2.2 Survey Development

The survey was developed by the research team, consisting of two clinically trained researchers and two urologists, and was based on their clinical experience. In a structured consensus meeting, an independent urologist and four specialized continence nurses filled in the questionnaire and were consulted for feedback, but no adjustments were necessary. The questionnaire comprises of 12 multiple‐choice questions regarding the field of work, work experience, and the decision‐making processes regarding catheterization method and other treatment options. The survey was constructed in Castor EDC, an open‐source online data capture application. The invitation contained an explanation of the study and a direct hyperlink to the survey. All data was collected anonymously, and informed consent was implied by the completion of the survey.

The primary outcome was to explore and compare the urinary catheter decision‐making process between different Dutch healthcare providers.

### 2.3 Statistical Analysis

All categorical variables are presented as frequencies and percentages. Categorical variables are analyzed using the chi‐square test and Fisher's exact test. Questionnaires with missing data were excluded from the data set. Statistical analyses were performed using IBM SPSS Statistics version 25 with a critical significance level of *p* < 0.05. The study followed the Strengthening the Reporting of Observational Studies in Epidemiology (STROBE) reporting guideline [14].

## 3 Results

The survey was sent to 300 healthcare providers; a total of 164 healthcare providers participated, of whom 108 completely filled in the questionnaire (response rate 36%). Of the respondents, 49 (45%) were (continence) nurses, 46 (43%) were urologists (in training), eight (7%) were PAs, and five (5%) were rehabilitation doctors (in training). Eighty‐seven percent worked in an academic or non‐academic hospital, and 34% had more than 15 years of experience in the field. Eighty‐five percent preferred CIC over IDC as a catheterization method for a patient, no healthcare provider preferred an IDC over CIC. An overview of the baseline characteristics is provided in Table 1.

**Table 1:** Baseline characteristics.

	*N* = 108	%
Job		
(Continence)nurse	49	45
Urologist	46	43
Physician assistant	8	7
Rehabilitation doctor	5	5
Workplace		
Hospital	94	87
Rehabilitation center	14	13
Years of experience (years)		
0−5	33	31
5−10	29	27
10−5	9	8
More than 15	37	34
Most often treated in the outpatient clinic		
IDC	60	55
CIC	48	45
Personal preference		
IDC	0	0
CIC	92	85
No specific preference	16	15
Frequency seeing patients with an IDC		
Daily	9	8
Weekly	42	39
Monthly	45	41
Yearly	7	6
Less than 1x per year	5	5
Frequency seeing patients who perform CIC		
Daily	6	6
Weekly	60	55
Monthly	36	33
Yearly	3	3
Less than 1x per year	3	3

Abbreviations: CIC = clean intermittent catheterization, IDC = indwelling catheter.

### 3.1 Healthcare Providers

**Figure 1:**
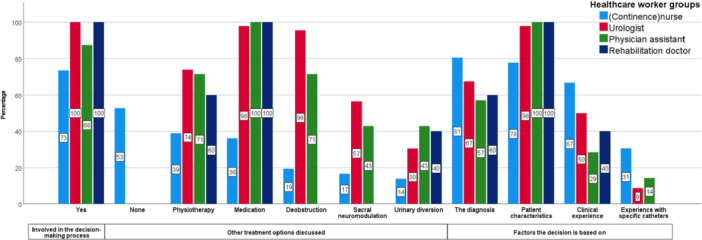
Answers of healthcare workers to the questions: “Are you involved in the decision‐making process?,” “What other treatment options do you discuss?,” and “On what factors do you base your decision what type of catheterization to prescribe?”

Seventy‐three percent of (continence)nurses and 87% of PAs were involved in the decision‐making process regarding the choice of catheterization method. All doctors were involved. Fifty‐three percent of the nurses involved in the decision‐making did not discuss any other treatment options besides the catheterization method. Urologists discussed physiotherapy, medication, deobstruction in male patients, and sacral neuromodulation significantly more often compared to (continence)nurses (*p *= 0.008, *p* < 0.001, *p* < 0.001, *p* = 0.001, respectively). PAs discussed deobstruction significantly more often compared to (continence)nurses (*p *= 0.015). (Continence)nurses based their decision significantly more on their experience with a specific catheter than urologists (*p *= 0.033). Urologists based their decision significantly more often on patient characteristics than (continence)nurses (*p *= 0.04). The data per healthcare provider group is shown in Figure 1.

### 3.2 (Continence)nurses and Urologists in a Hospital

Because most urinary catheters are prescribed in the urology department of hospitals, we compared the decision‐making processes of urologists and (continence)nurses working in a urology department. Urologists were significantly more involved in the decision‐making process compared to (continence)nurses (*p* < 0.001). Urologists discussed all options, apart from urinary diversion, significantly more compared to (continence)nurses (*p* < 0.001 in all).

**Figure 2:**
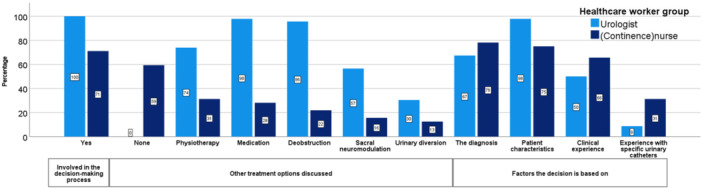
Answers of urologists and (continence) nurses working in the urology department to the questions: “Are you involved in the decision‐making process?,” “What other treatment options do you discuss?,” and “On what factors do you base your decision what type of catheterization to prescribe?”

Urologists were more likely to base their decision which catheterization method to prescribe on patient characteristics compared to (continence)nurses (*p *< 0.001). In contrast, (continence)nurses based their decision significantly more often on their experience with specific catheters (*p *= 0.02). The data comparing urologists with (continence)nurses working at the urology department are shown in Figure 2.

### 3.3 Catheter Decision Aid

Eighty‐three out of the 108 healthcare providers think a validated catheter decision aid can improve the standard of care for catheter users. Nurses were the most positive group, with 84% thinking it can improve the standard of care for patients. Among PAs, support was limited—only one respondent (13%) believed it would improve patient care. The data on a catheter decision aid is shown in Table 2.

**Table 2:** Perceived added value of a catheter decision aid among healthcare providers. Answers of healthcare providers to the question: “Do you think that a catheter decision aid could improve care for catheter users?”

(Continence)nurse (*n* = 49)		Urologist (*n* = 46)	Physician assistant (*n* = 7)	Rehabilitation doctor (*n* = 5)	Total (*n* = 108)
Catheter decision‐making aid					
Yes	41 (84)	32 (70)	1 (13)	3 (60)	83 (76)
No	8 (16)	14 (30)	7 (87)	2 (40)	26 (24)

## 4 Discussion

In this study, we identified the decision‐making process of healthcare providers regarding the type of catheterization. The majority of the healthcare providers in this study preferred CIC over IDC when a patient is in need of a urinary catheter. Nurses were involved less in the decision‐making process, and discussed other treatment options besides urinary catheterization less. Most healthcare providers based the choice of catheterization method on patient characteristics.

As stated before, the vast majority of healthcare providers preferred CIC over an IDC when urinary catheterization is indicated. This is in line with the guidelines, which state that CIC is the golden standard in bladder management [3]. However, in the clinic, the majority of the healthcare providers consult patients with an IDC more frequently. This can be due to a number of reasons. First, to perform CIC a patient needs an adequate manual dexterity and cognitive function, both of which may decline with increasing age. The likelihood of catheterization increases with age, leading to a greater number of people receiving an IDC [13]. Second, patients can perceive the CIC procedure as intimidating, with significant physical, psychological, and emotional impact [15], thus opting for an IDC. Third, due to time constraints and limited healthcare personnel, healthcare providers may be more inclined to recommend an IDC [15].

Regarding the impact of advancing age on the ability to perform CIC, Hentzen et al. and Parsons et al. reviewed patients who used CIC to empty their bladder [16, 17]. In these cohorts, patients aged over 65 generally demonstrated the ability to perform CIC effectively, with success rates of 84% and 82%, respectively. These studies show that older patients are also able to perform CIC successfully and, thus, should not be deprived of the possibility to do so.

The majority of (continence)nurses working in urology departments do not discuss other treatment options. In addition, (continence)nurses in these departments discuss all treatment options significantly less compared to urologists, except from urinary diversion. As a result, patients consulted by a (continence)nurse may receive less comprehensive information when making a decision about their bladder management and thus might not receive the optimal treatment.

Although primary consultations are typically conducted by a urologist, the follow‐up care is commonly managed by nurses, general practitioners, or district nurses. In addition, with the increasing demand for healthcare and the growing use of urinary catheters, nurses are likely to take on a more significant role in primary decision‐making. Educating nurses about the advantages, disadvantages, and psychosocial aspects of various treatment options is important. In a recent systematic review, Alex et al. showed that upskilling nurses and improving their confidence to deliver patient‐centered catheter care led to better outcomes in patients with long‐term indwelling urinary catheters [18].

Shared decision making (SDM) is most applicable in preference‐sensitive situations, where multiple, equally efficacious treatment options exist, and the treatment option is based on patients' preferences [19]. The process of deciding which catheter to prescribe is particularly well‐suited to SDM. SDM has the potential to increase patient knowledge, health outcomes, and reduce unwarranted variation in care and costs [20]. The decision‐making process in urinary catheters is very suitable for SDM. During the decision‐making process, medical risks and psychosocial aspects of the treatment options should be discussed. Additionally, it is important to discuss all treatment options, ensuring that patients understand why certain options may not be appropriate for them. Implementing SDM in the decision‐making process of catheterization method and relevant bladder management provides the patient with a sense of autonomy and may enhance satisfaction with their bladder management [21].

Although promoted by health policies and research, the implementation of SDM has proven to be a challenging task [22]. On one hand, healthcare providers face barriers such as time constraints, lack of skills, and the healthcare provider's attitude toward SDM. On the other hand, patients face barriers such as a lack of knowledge and the nature of their relationship with healthcare providers. Much research has been done on how to overcome these barriers and successfully implement SDM. The training of clinicians and using patient decision aids have been suggested as potential solutions and foster the implementation of SDM [23, 24].

SDM will only be fully integrated if healthcare providers view SDM as a routine practice and an essential component of safe, effective, and compassionate healthcare. Therefore, healthcare providers need extensive training to overcome the lack of knowledge and communication problems [22]. Incorporating SDM in the curricula of medical and nursing schools will better prepare future healthcare providers to engage in SDM [25, 26].

Decision aids can lead to an increase in knowledge, improve accurate risk perceptions, and promote an active role in decision‐making [27]. In other urological conditions, including renal, bladder, and prostate cancer, decision aids have shown to decrease decisional regret and improve patient satisfaction with decisions, quality of decision‐making, and trust between provider and patient [28, 29, 30].

In a recent survey study of 117 NLUTD care providers, 88% rated the need for an NLUTD decision aid as strongly needed or somewhat needed. Seventy‐eight percent expressed that they would use an NLUTD decision aid if available [31]. These results are comparable to ours and indicate the demand for a bladder management decision aid.

Future studies should focus on implementing SDM and the development of a decision aid. It is important that both healthcare providers and patients are involved to achieve the optimal usability and adoption of the decision‐aid in clinical practice. Thereby, healthcare costs and the impact on the environment of the treatment options should also be included in the decision aid; this could be done by encouraging the use of reusable catheters for CIC.

This study has some limitations. First, the low response rate and imbalance between healthcare providers may limit the generalizability of the findings. Nonetheless, these values remain within the range observed in survey studies in the field of urology [32, 33]. Second, we did not include GPs in the study. Although GPs account for a substantial part of IDCs prescribed, they do not prescribe or manage CIC [34]. Third, the survey was not validated, limiting the generalizability and increasing potential bias. Another limitation is the absence of a sample size calculation. As the goal of this study was to explore differences in decision‐making among healthcare workers, rather than to identify statistical differences, we did not perform a sample size calculation.

## 5 Conclusion

In this study, we observed differences in the decision‐making processes among healthcare providers prescribing urinary catheters. Healthcare providers are involved to varying degrees in the decision‐making process and base their decisions on various factors. To optimize bladder management treatment, the decision‐making process should be guided by SDM. Implementing SDM promotes patient autonomy and can increase patient satisfaction with their bladder management. To minimize variability among healthcare providers and facilitate SDM, comprehensive training supplemented with a validated decision aid may present a promising solution. A decision aid can help inform patients and clarify the decision‐making process, enabling better collaboration between patients and healthcare providers in selecting the most appropriate type of bladder management.

## Ethics Statement

This research received approval from the Medical Ethical Review Committee (MEC‐2024‐0304).

## Consent

The study data were collected anonymously, and informed consent was implied by completing the survey.

## Conflicts of Interest

The authors declare no conflicts of interest.

## Data Availability

The data supporting the findings of this study are available upon reasonable request to the corresponding author.
